# Sex as predictor for achieved health outcomes and received care in ischemic stroke and intracerebral hemorrhage: a register-based study

**DOI:** 10.1186/s13293-018-0170-1

**Published:** 2018-03-07

**Authors:** Carl Willers, Ingrid Lekander, Elisabeth Ekstrand, Mikael Lilja, Hélène Pessah-Rasmussen, Katharina S. Sunnerhagen, Mia von Euler

**Affiliations:** 1 Department of Clinical Science and Education, Södersjukhuset, Karolinska Institutet, Sjukhusbacken 10, 118 61 Stockholm, Sweden; 2 Karolinska Institutet Stroke Research Network at Södersjukhuset, Stockholm, Sweden; 3 Ivbar Institute AB, Stockholm, Sweden; 4 Medical Management Centre, Karolinska Institutet, Stockholm, Sweden; 5 Department of Health Sciences, Lund University, Lund, Sweden; 6 Department of Public Health and Clinical Medicine, Unit of Research, Education and Development, Östersund, Umeå University, Umeå, Sweden; 7 Department of Neurology and Rehabilitation Medicine, Skåne University Hospital, Lund, Sweden; 8 Department of Clinical Sciences, Lund University, Lund, Sweden; 9 Institute of Neuroscience and Physiology, Rehabilitation Medicine, University of Gothenburg, Gothenburg, Sweden; 10 Center for Gender Medicine, Department of Medicine Solna, Karolinska Institutet, Stockholm, Sweden

**Keywords:** Stroke, Sex, Health outcomes, Resources, Utilization, Epidemiology

## Abstract

**Background:**

Differences in stroke care and health outcomes between men and women are debated. The objective of this study was to explore the relationship between patients’ sex and post-stroke health outcomes and received care in a Swedish setting.

**Methods:**

Patients with a registered diagnosis of acute intracerebral hemorrhage (ICH) or ischemic stroke (IS) within regional administrative systems (ICD-10 codes I61* or I63*) and the Swedish Stroke Register during 2010–2011 were included and followed for 1 year. Data linkage to multiple other data sources on individual level was performed. Adjustments were performed for age, socioeconomic factors, living arrangements, ADL dependency, and stroke severity in multivariate regression analyses of health outcomes and received care. Health outcomes (e.g., survival, functioning, satisfaction) and received care measures (regional and municipal resources and processes) were studied.

**Results:**

Study population: 13,775 women and 13,916 men. After case-mix adjustments for the above factors, we found women to have higher 1-year survival rates after both IS (OR_female_ = 1.17, *p* < 0.001) and ICH (OR_female_ = 1.65, *p* < 0.001). Initial inpatient stay at hospital was, however, shorter for women (*β*_female, IS_ = − 0.05, *p* < 0.001; *β*_female, ICH_ = − 0.08, *p* < 0.005). For IS, good function (mRS ≤ 2) was more common in men (OR_female_ = 0.86, *p* < 0.001) who also received more inpatient care during the first year (*β*_female_ = − 0.05, *p* < 0.001).

**Conclusions:**

A lower proportion of women had good functioning, a difference that remained in IS after adjustments for age, socioeconomic factors, living arrangements, ADL dependency, and stroke severity. The amount of received hospital care was lower for women after adjustments. Whether shorter hospital stay results in lower function or is a consequence of lower function cannot be elucidated. One-year survival was higher in men when no adjustments were made but lower after adjustments. This likely reflects that women were older at time of stroke, had more severe strokes, and more disability pre-stroke—factors that make a direct comparison between the sexes intricate.

**Electronic supplementary material:**

The online version of this article (10.1186/s13293-018-0170-1) contains supplementary material, which is available to authorized users.

## Background

In Sweden as in many other Western countries, women and men suffer stroke to the same extent, although women in general have their strokes later in life [1–3]. Several studies have shown that women in general have more severe symptoms at arrival to hospital, a worse prognosis, and are less likely to return to home and independent living, but there are also conflicting data [4–9]. Whether the patient’s sex affects aspects of stroke care has been investigated, but findings do not consistently point in one direction [4, 8, 9]. Studies designed to clarify unresolved issues on this topic are warranted.

Health equity is generally seen as the absence of health disparities and care on equal prerequisites given potentially differing needs [10]. Sex as a statistically significant predictor for health outcomes after stroke should not be the case if Swedish stroke care is to be viewed as equal. In stroke, clinical presentation is similar for men and women with some potential exceptions [11–14], and recommended treatments are overall the same [3].

The objective of this study was to analyze post-stroke health outcomes and received care in relation to sex for ischemic stroke (IS) and intracerebral hemorrhage (ICH), respectively.

## Methods

### Study population and data sources

Patients registered in both the regional patient administrative systems (PAS), inpatient with ICD-10 codes I61* (ICH, not including SAH) or I63* (IS), and in the Swedish Stroke Register (a national registry of all strokes with a coverage rate of > 90% [15, 16]) from 1 January 2010 to 31 December 2011 were included in this study. Stroke patients identified only with an I64* diagnosis were not included (1.4% of the total). Seven Swedish regions delivered data, covering ~ 65% of the Swedish population.

PAS data include all clinical factors (diagnoses, information on prior stroke, and comorbidity) and are available from hospitals as well as from outpatient care. Through the unique personal identification numbers, patient-level data were linked between multiple data sources: the Swedish Stroke Register (containing information on stroke severity, functional status, treatment, patient-reported data), Statistics Sweden (socioeconomic status, survival), the National Board of Health and Welfare (municipality services), and the Swedish Social Insurance Agency (sick leave, disability pension).

### Study variables

The research database consisted of a wide range of health outcome and resource measures. Identification of key study variables and factors influencing the outcome (case-mix factors) were based on the available literature and clinical expertise of the extended research group (Sveus, https://www.sveus.se/) representing regions, patient organization, specialists, quality registries, and Ivbar Institute (R&D company). Selected study variables are presented below (key data source in parentheses).

Indicators of achieved health outcomes:

- One-year all-cause mortality (Statistics Sweden)
- One-year recurrent stroke (> 28 days after the first stroke, PAS)
- Good functional status after 1 year—approximated modified Rankin Scale (mRS) ≤ 2 (patient-reported, computed in accordance with Eriksson et al. [17], Swedish Stroke Register)
- Good perceived general health 1 year after stroke (patient-reported, Swedish Stroke Register)
- Return to formal full-time work ability 1 year post-stroke (Swedish Social Insurance Agency)

Indicators of received care—resource use:

- Initial inpatient stay (PAS)
- Inpatient stay first year post-stroke (PAS)
- Outpatient visits first year post-stroke (PAS)
- Net days of sick leave/disability pension first year post-stroke for patients in general employment age (< 66 years, Swedish Social Insurance Agency)
- Added hours of home help services first year post-stroke (National Board of Health and Welfare)
- Proportion of patients transferred to special housing first year post-stroke (not staying in special housing before stroke onset, National Board of Health and Welfare)

Indicators of received care—process measures:

- Thrombolysis (only IS, Swedish Stroke Register)
- Thrombectomy (only IS, Swedish Stroke Register)
- High/very high three-month satisfaction (patient-reported, Swedish Stroke Register)

### Statistical analysis

Results are presented through descriptive statistics of the study population and as case-mix adjusted regression analysis output.

#### Stratified analysis of crude values

Potential differences in observed values between sexes were investigated by stratifying the study variable outcomes on women and men respectively, calculating 95% confidence intervals (CI). Stratification shows the crude (unadjusted) levels, without adjusting for factors that may systematically differ between sexes.

#### Regression analysis

Potential sex differences were analyzed through regression analysis. Firstly, sex was used as sole case-mix factor in univariate regression analyses. Secondly, regression analyses were performed with the following independent variables:

- Sociodemographic factors (in addition to sex): age, level of education, living alone, marital status, and born in a country being a member of the European Union in 2010 (EU-27).
- Health profile at baseline: living arrangements, dependency for activities of daily life (ADL), prior stroke registered in medical records within the last 2 years, and consumption of inpatient care the year before stroke (approximating previous and current comorbidity).
- Stroke characteristics: level of consciousness at hospital arrival. Due to a coverage rate below 50%, the National Institutes of Health Stroke Scale (NIHSS) was not used. IS and ICH were analyzed separately.

Health outcomes, process measures, and transfer to special housing were modeled as binary outcomes with logistic regression (regression coefficients presented as odds ratios, OR). Resource dimensions were treated as count variables with negative binomial regression (coefficients presented as log count, LC) except added home help service which was modeled as a continuous variable with linear regression (home help service analysis included only patients who were not living in special housing pre-stroke). Adjustment for age differences in regression analysis was performed with 10-year age groups. A 95% significance level was used (*p* < 0.05). When calculating response rates in patient-reported outcome data, only survivors at 3 and 12 months, respectively, were included. STATA v.13.0 was used for statistical analyses.

## Results

**Fig. 1:**
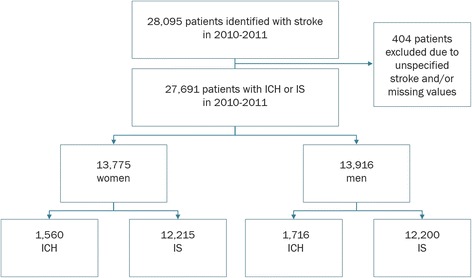
Study population

For 2010 and 2011, 27,691 patients were identified with a registered stroke, specified as IS or ICH—13,775 (49.7%) women and 13,916 (50.3%) men. Of these, 24,415 (88.2%) had IS and 3276 (11.8%) had ICH. Figure 1 presents the study population divided by sex and stroke subtype.

Within 1 year after stroke, 5707 (23.4%) of the IS patients had died (26.8% of women, 19.9% of men) and 1323 (40.3%) of the ICH patients (42.0% of women, 38.9% of men).

### Baseline characteristics

Women were older at the time of stroke, on average 5.1 and 5.8 years for IS and ICH, respectively. A smaller proportion of the female patients had higher education level than comprehensive school compared to the male patients. More women lived alone at the time of stroke, and a larger proportion of the women were widowed. A larger proportion of the women lived in special housing at stroke onset, and ADL dependency was overall more common in female patients. The proportion of patients with prior registered stroke was equally distributed between sexes whilst the prevalence of atrial fibrillation and hypertension was higher in women except for atrial fibrillation in ICH patients (no difference). Smoking was not included in order not to reduce the sample size (coverage of < 90%). Women presented with more severe strokes, and more women than men were unconscious at arrival (Table 1).

**Table 1:** Descriptive statistics of study population, at baseline. 95% CI

Category	Variable	Ischemic stroke			Intracerebral hemorrhage		
		All	Women	Men	All	Women	Men
Sociodemographic profile	Number of patients (%)	24,415	12,215 (50.0%)	12,200 (50.0%)	3276	1560 (47.6%)	1716 (52.4%)
	Age in years (mean)	76.5 (76.4; 76.7)	79.1 (78.8; 79.3)	74.0 (73.8; 74.2)	73.9 (73.4; 74.3)	76.9 (76.2; 77.5)	71.1 (70.5; 71.8)
	Highest level of education (distribution, %)						
	Elementary	46.7	51.7	41.7	43.4	48.6	38.8
	High school	36.5	34.2	38.9	36.8	34.4	39.0
	College/university	16.8	14.2	19.4	19.7	17.0	22.2
	Marital status (distribution, %)						
	Married	42.4	30.1	54.7	43.8	33.6	53.1
	Unmarried	11.1	8.6	13.6	14.4	10.5	17.9
	Divorced	16.7	16.2	17.2	17.5	16.6	18.3
	Widowed	29.8	45.1	14.5	24.4	39.4	10.7
	Born outside the EU (%)	4.8 (4.5; 5.1)	4.4 (4.0; 4.7)	5.3 (4.9; 5.7)	6.2 (5.4; 7.0)	5.4 (4.3; 6.6)	6.9 (5.7; 8.1)
	Living alone (%)	51.3 (50.7; 51.9)	64.7 (63.9; 65.6)	37.8 (37.0; 38.7)	49.4 (47.7; 51.1)	61.1 (58.6; 63.5)	38.7 (36.4; 41.0)
	Living arrangements (distribution, %)						
	Living at home, no home help services	71.3	62.9	79.8	71.3	61.8	80.1
	Living at home, with home help services	19.0	24.8	13.2	16.6	23.0	10.7
	Special housing	9.7	12.4	6.9	11.5	14.9	8.3
Medical history	ADL dependency (%)	11.4 (11.0; 11.8)	13.8 (13.2; 14.4)	9.0 (8.5; 9.6)	12.1 (10.9; 13.2)	15.9 (14.0; 17.7)	8.7 (7.4; 10.1)
	Prior stroke (− 2 years) (%)	6.9 (6.5; 7.2)	6.8 (6.3; 7.2)	6.9 (6.5; 7.4)	6.5 (5.7; 7.3)	7.3 (6.0; 8.6)	5.8 (4.7; 6.9)
	Inpatient care year − 1 (mean days)	5.2 (5.0; 5.3)	5.6 (5.4; 5.8)	4.7 (4.5; 5.0)	5.7 (5.2; 6.3)	6.6 (5.8; 7.4)	5.0 (4.3; 5.6)
	Atrial fibrillation (%)	31.0 (30.4; 31.6)	34.2 (33.3; 35.0)	27.8 (27.0; 28.6)	21.6 (20.2; 23.0)	20.6 (18.6; 22.6)	22.6 (20.6; 24.5)
	Hypertension (%)	64.7 (64.1; 65.3)	67.5 (66.6; 68.3)	62.0 (61.1; 62.8)	59.9 (58.2; 61.6)	62.6 (60.2; 65.0)	57.5 (55.2; 59.9)
Stroke severity	Level of consciousness at hospital arrival (distribution, %)						
	Conscious	85.9	83.0	88.8	60.3	57.6	62.7
	Indolent	10.9	13.0	8.8	22.1	23.7	20.7
	Unconscious	3.2	4.0	2.4	17.6	18.7	16.7

**Fig. 2:**
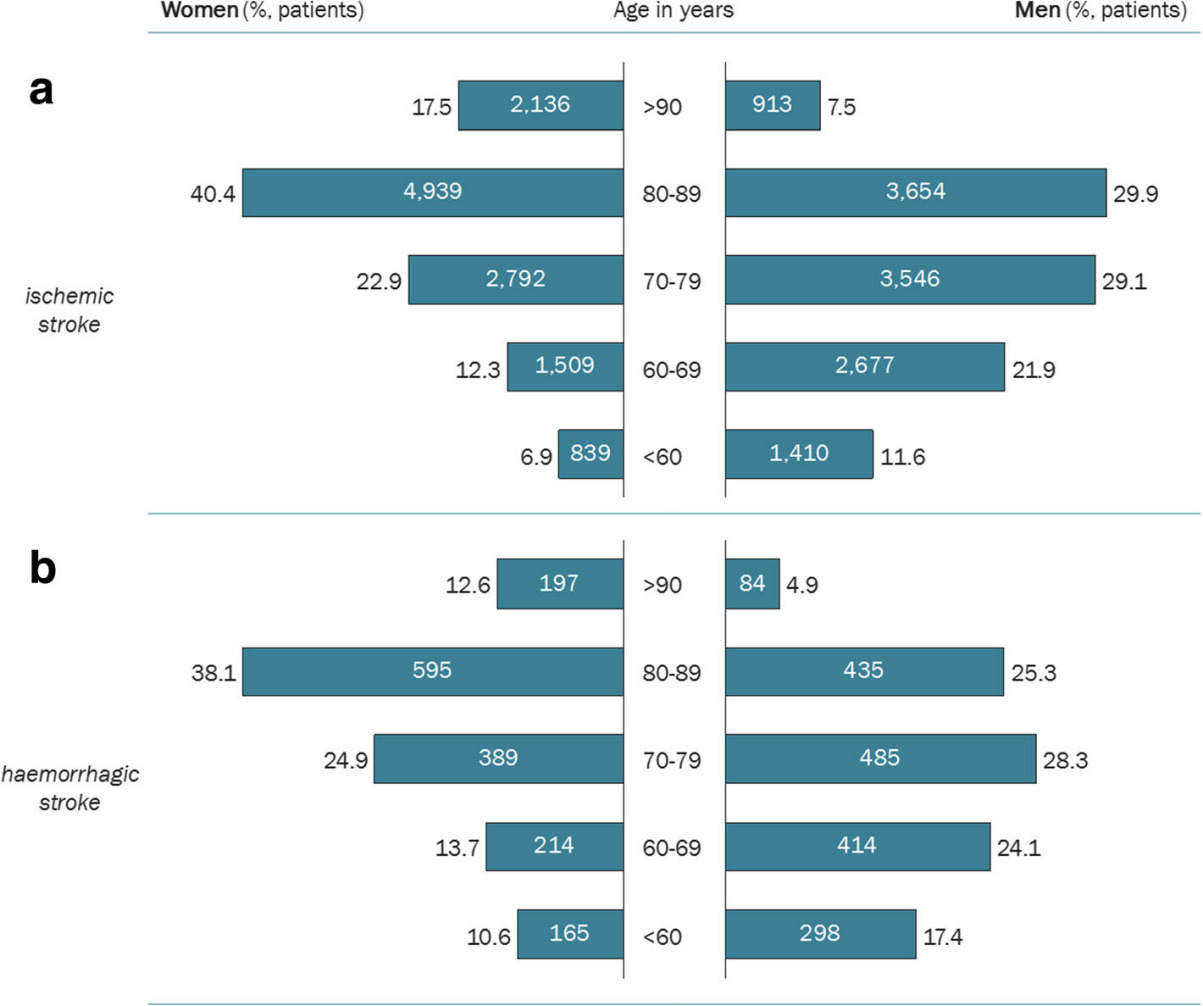
Age distribution for women and men with IS (**a**) and ICH (**b**)

Women dominated the two eldest groups (80–89 years and > 90 years) irrespective of stroke subtype (Fig. 2). On the contrary, men dominated the three younger age groups; male stroke patients below 70 years of age were almost double the quantity of female stroke patients below 70 years.

### Stratified analysis of crude values

Crude values imply that women with IS had lower levels of 1-year survival, 1-year good functioning (approximated mRS ≤ 2), and 1-year patient-reported good general health and fewer first-year outpatient visits but higher levels of net sick leave and added home help service hours. A larger proportion of women were transferred to special housing during the first year (in addition to a larger proportion already living in special housing before stroke onset), and a smaller proportion showed high 3-month patient-reported satisfaction. The proportion receiving reperfusion treatment with either thrombolysis or thrombectomy was lower in women than in men. For ICH, women were found to have worse 1-year functioning, shorter initial inpatient stay, fewer outpatient visits, and an almost twice as high proportion transferred to special housing compared with men. Crude values stratified by sex and stroke subtype can be found in Additional file 1: Table S1.

### Regression analysis

**Fig. 3:**
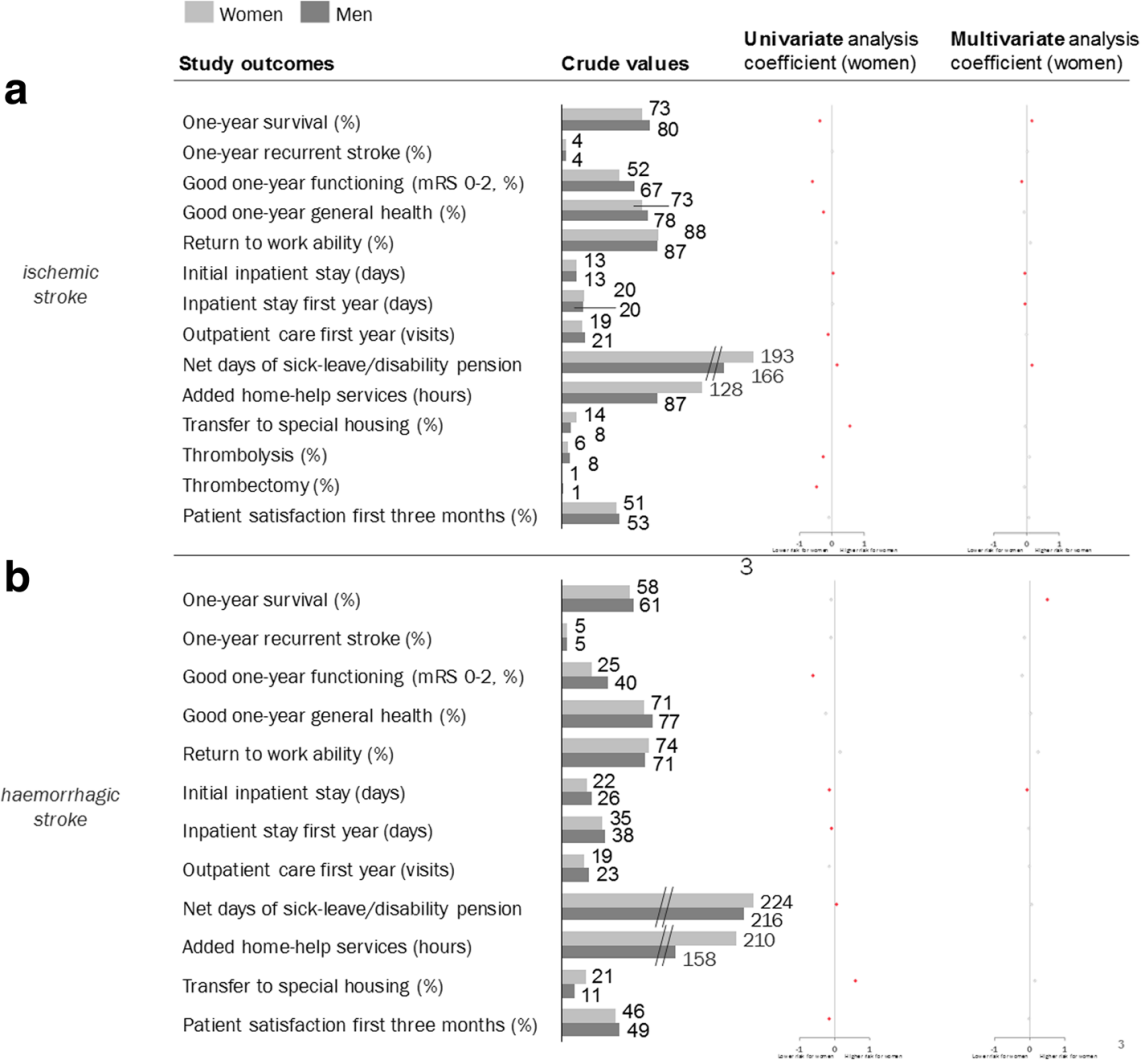
Regression analyses for IS and ICH, univariate and multivariate. Coefficients presented for female sex (male as reference). Red mark indicates statistically significant deviation on 95% significance level

Results from the univariate and multivariate regression analysis are presented in Table 2 and Fig. 3. Information on analysis population sizes can be found in Additional file 1: Table S2. For the multivariate regression analysis on IS patients, women had higher 1-year survival, OR 1.17 (1.11; 1.24), and lower proportion with good 1-year functioning, OR 0.86 (0.82; 0.89). Initial and first-year inpatient care levels were lower for women, LC − 0.05 and confidence intervals (− 0.07; − 0.03) and (− 0.08; − 0.03), respectively—the difference in initial inpatient care lasted throughout the first year after stroke. The level of sick leave was higher for women, LC 0.17 (0.13; 0.21). A lower proportion of women were transferred to special housing compared to men after ischemic stroke, OR 0.97 (0.94; 0.99). Fewer study variables differed between men and women in ICH than in IS, but women had higher 1-year survival, OR 1.65 (1.36; 1.99), and shorter initial inpatient stay, LC − 0.08 (− 0.13; − 0.03), after ICH (Table 2).

**Table 2:** Regression analyses for IS and ICH, univariate and multivariate. Coefficients presented for female sex (male as reference) with 95% CI

	Sex as predictor for IS				Sex as predictor for ICH			
	Univariate		Multivariate		Univariate		Multivariate	
	Coefficient	95% CI	Coefficient	95% CI	Coefficient	95% CI	Coefficient	95% CI
1-year survival (OR)	0.69	(0.66; 0.72)***	1.17	(1.11; 1.24)***	0.91	(0.77; 1.07)	1.65	(1.36; 1.99)***
1-year recurrent stroke (OR)	1.01	(0.93; 1.10)	1.02	(0.94; 1.11)	0.91	(0.71; 1.16)	0.86	(0.64; 1.16)
Good 1-year functioning (approximated mRS 0–2) (OR)^a^	0.55	(0.52; 0.59)***	0.86	(0.82; 0.89)***	0.64	(0.45; 0.91)*	1.05	(0.68; 1.61)
Good 1-year general health (OR)^a^	0.77	(0.69; 0.85)***	0.93	(0.83; 1.04)	0.78	(0.58; 1.06)	1.03	(0.83; 1.28)
Return to formal full-time work ability (OR)	1.14	(0.92; 1.42)	1.13	(0.91; 1.41)	1.15	(0.90; 1.50)	1.28	(0.93; 1.75)
Initial inpatient stay (LC)	0.03	(0.01; 0.05)**	-0.05	(− 0.07; − 0.03)***	− 0.15	(− 0.21; − 0.09)***	− 0.08	(− 0.13; − 0.03)**
Inpatient days first year (LC)	0.02	(− 0.01; 0.05)	− 0.05	(− 0.08; − 0.03)***	− 0.09	(− 0.16; − 0.01)*	− 0.03	(− 0.11; 0.05)
Outpatient visits first year (LC)	− 0.12	(− 0.14; − 0.10)***	0.00	(− 0.03; 0.02)	− 0.15	(− 0.30; − 0.00)*	− 0.01	(− 0.17; 0.14)
Net days of sick leave/disability pension (LC)	0.16	(0.11; 0.20)***	0.17	(0.13; 0.21)***	0.05	(0.02; 0.09)**	0.05	(− 0.01; 0.10)
Added home help services (hours)	40.8	(20.9; 60.7)**	− 1.6	(− 25.7; 22.4)	34.9	(− 36.8; 106.5)	− 3.9	(− 88.0; 80.2)
Transfer to special housing (OR)	1.73	(1.63; 1.83)***	0.97	(0.94; 0.99)*	1.82	(1.54; 2.15)***	1.16	(0.90; 1.49)
Thrombolysis (OR)	0.76	(0.64; 0.91)***	1.07	(0.98; 1.16)				
Thrombectomy (OR)	0.62	(0.53; 0.72)***	0.94	(0.80; 1.10)				
High three-month patient satisfaction (OR)	0.91	(0.83; 1.00)	1.06	(0.98; 1.15)	0.86	(0.76; 0.97)*	0.97	(0.85; 1.11)

Multivariate regression analysis adjusted for (in addition to sex) age, education level, living alone, marital status, born in a EU-27 country, living arrangements, ADL dependency, prior stroke according to medical records (last 2 years), consumption of inpatient care the year before stroke (approximating previous and current comorbidity), and level of consciousness at hospital arrival

*p < 0.05; **p < 0.005; ***p < 0.001

^a^Coverage rate of outcome variable amounted to less than 80% for the IS study population

Some study variables differed between sexes in the univariate as well as in the multivariate analysis, but with opposite effects of the patient’s sex; 1-year survival for both stroke subtypes, and for IS, initial and first-year inpatient stay as well as transfer to special housing. The patient’s sex affected some variables in the same direction within both analyses, but with different sizes of effect—initial inpatient stay (ICH), 1-year functioning, and sick leave (IS).

Response rates to patient-reported questionnaires in the Swedish Stroke Register after IS were lower for women at 3 months’ follow-up but not at 12 months—84.9% (84.2; 85.6) and 68.8% (67.8; 69.8), respectively—compared to men with response rates 86.4% (85.8; 87.1) and 70.1% (69.2; 71.0), respectively for IS. The patterns were similar for ICH patients.

## Discussion

In our study, women were found to have significantly better survival in multivariate analyses, correcting for factors such as stroke severity, risk factors, and age at stroke, whilst men showed higher 1-year survival in univariate analyses. Thus, even though more women than men died after having a stroke, the risk for patients of the same age, with similar risk factors to die after a stroke of similar severity, was higher for men. Women’s all-cause mortality after stroke has been put forward as lower when adjusting for other relevant factors [18], but there are also results opposing that [19] as well as dismissing any significant differences [20]. Variations in the findings on sex differences between previous studies [21–25] may not only reflect differences in the population but also differences in the adjustments performed. Such general differences in statistical approach have also been pointed out in recently published American guidelines [14]. Within this study, adjustments made involved a broad range of relevant factors, potentially implying higher accuracy than some of the previous comparisons. With similar  adjustments made, a Danish study also showed higher stroke survival in women [26]. For studying factual outcome, a univariate analysis may have advantages, but for studying health care delivery from a health equity point of view, a multivariate adjustment could be regarded as more appropriate. For example, in univariate analysis, a higher proportion of men were found to receive reperfusion treatment, a treatment choice that is affected by age, pre-stroke function, and onset-to-hospital arrival time [27]; in multivariate analyses (correcting for age and living conditions), no differences remained.

We found the rates of good 1-year functioning after IS to be lower in women, in line with previous studies [21–23]. There are also studies pointing to no significant differences [24, 25]. Any such differences could be attributable to sex bias in the diagnosis and treatment or to the biological differences in the disease. Stroke severity has been shown to be worse in women [28]. Previous research points to the differences in muscle strength being a confounding factor for function and ADL [29], and neurological damage to the brain may have a different impact in women and men [30]. Functional status was calculated from patient-reported measures which could potentially vary systematically between men and women.

We found women to have significantly shorter initial inpatient stay (IS and ICH) and total first-year inpatient stay (IS)—potentially due to that more women were living in special housing before and after stroke. Several studies have shown equal rehabilitation levels [31], implying that women are less responsive to rehabilitation, and hence in larger need of it [32].

Women were transferred to special housing after IS to a lower degree than men. This should however be expected as the proportion of women already living in special housing at time of stroke onset was almost the double. These higher proportions possibly affect the sex differences observed regarding inpatient care and stroke-specialized rehabilitation levels as both are expected to be lower in these patients.

Women are generally older than men at the time of stroke onset. Also, women have a longer life expectancy. In Sweden 2011, the average lifespan in women was 83.7 and 79.8 years in men [33]. For the last hundred years, women have in general been 3 years younger than their spouse [34]. Consequently, a higher rate of widowed women live in single households which also implies different prerequisites, different need of special housing, treatment pathways, and expected health outcomes. Stroke severity, living conditions, and marital status were significant predictors for several post-stroke outcomes in the multivariate analysis.

Rate of return to formal full-time work ability (patients < 66 years) did not differ between sexes. However, women had approximately 16% more net days of sick leave/disability pension after IS. In the general population, women had 34% more sick leave (Sweden 2010) [35]. Thus, sick leave differences should probably be viewed as originating from other societal structures and not as results from stroke care.

### Strengths and limitations

Given the study’s base in registry data, it was not possible to fully assure that the patient sample studied was unbiased. However, high coverage rates of the Swedish national quality register [15, 16] and the administrative systems together with a two-year incidence population (2010–2011) covering a majority of the Swedish stroke cases imply a high validity of the study sample and possible conclusions. Coverage rate was limited for some patient-reported variables (85.7% and 69.5% for 3- and 12-month approximated mRS, respectively, in IS patients) but very high for most administrative data.

There may still be residual confounding present. Some additional factors are known to have an impact on the outcomes and burden of stroke respectively, including stroke severity (NIHSS), metabolic (e.g., smoking, diet), and environmental factors (e.g., lead exposure, air pollution) [36]. Due to low coverage of NIHSS, level of consciousness (excellent coverage) was used, as the correlation to functional status, and survival has been shown to be high [37]. Post-hoc correction to account for the multiple tests performed between sexes has not been presented in this paper. However, a Bonferroni correction would imply a new cutoff for statistical significance equivalent to *α*/*n* = 0.05/14 = 0.0036, still leaving all differences found after multivariate adjustments significant except for initial inpatient stay in ICH patients.

Information on informal care such as support at home from family/spouse would have added an important perspective. Furthermore, municipal health care data were not available. It is worth noting that differences in inpatient care between women and men may be due to other reasons than stroke care. There is a diagnosis uncertainty in rates of recurrent stroke, related to differences in routines. To counter such systematic errors, recurrent stroke was defined as registered inpatient diagnosis at clinics managing acute stroke care. Potential systematic errors in registrations and medical records may be assumed to be equally distributed between sexes (possibly not applicable to patient-reported measures).

## Conclusions

Some differences in post-stroke care and health outcomes seem to exist between sexes. A lower proportion of women had a good functional status, a difference that remained in IS after adjustments for age, socioeconomics, living arrangements, ADL dependency, and stroke severity. Levels of received hospital care were lower for women. Whether shorter hospital stay results in lower function or is a consequence of lower function cannot be elucidated. Shorter inpatient stay for women may reflect the higher degree of women staying at special housing before and after stroke onset. One-year survival was higher in men when no adjustments were made but lower after adjustments, in line with women’s generally longer life expectancy. The difference after adjustments likely reflects that women were older at the time of stroke, had more severe strokes, and more disability pre-stroke—factors that make a direct comparison between the sexes intricate. A step towards a more equal stroke care in Sweden would possibly be to ensure that patients living in special housing receive the same levels of stroke-specialized rehabilitation as other stroke patients who remain in inpatient care.

## Additional file

Additional file 1: **Table S1.** Study outcomes stratified by sex, 95% CI. **Table S2.** Analysis population sizes and *p* values from multivariate regression analyses. (DOCX 29 kb)
