# Supplementary material for: Sex as predictor for achieved health outcomes and received care in ischemic stroke and intracerebral hemorrhage: a register-based study
Source: Biol Sex Differ. 2018 Mar 7;9:11. doi: 10.1186/s13293-018-0170-1 (PMC5842547; doi:10.1186/s13293-018-0170-1)
Supplement: Supplementary file 1 — Table S1. Study outcomes stratified by sex, 95% CI. Table S2. Analysis population sizes and p values from multivariate regression analyses. (DOCX 29 kb) [file 13293_2018_170_MOESM1_ESM.docx]

**Additional file 1**

**Table S1. Study outcomes stratified by sex.** 95% CI.

|  | | **Ischemic stroke** | | | **Intracerebral hemorrhage** | | |
| --- | --- | --- | --- | --- | --- | --- | --- |
| **Category** | **Study variable** | **All**  **(95% CI)** | **Women**  **(95% CI)** | **Men (95% CI)** | **All**  **(95% CI)** | **Women**  **(95% CI)** | **Men (95% CI)** |
| Health outcomes | One-year survival (%) | 76.6  (76.1; 77.2) | 73.2  (72.4; 74) | 80.1  (79.4; 80.8) | 59.6  (57.9; 61.3) | 58.0  (55.6; 60.5) | 61.1  (58.8; 63.4) |
|  | One-year recurrent stroke (%) | 4.1  (3.8; 4.4) | 4.2  (3.8; 4.7) | 4.1  (3.7; 4.5) | 5.2  (4.2; 6.2) | 5.1  (3.7; 6.6) | 5.3  (3.9; 6.7) |
|  | Good one-year functioning (approximated mRS 0-2, %) | 59.9  (59.1; 60.8) | 52.4  (51.1; 53.7) | 66.7  (65.5; 67.8) | 50.9  (48.0; 53.7) | 44.7  (40.5; 48.9) | 55.9  (52.1; 59.8) |
|  | Good one-year patient-reported general health (%) | 75.9  (75.1; 76.6) | 73.2  (72.1; 74.3) | 78.2  (77.2; 79.2) | 74.1  (71.6; 76.5) | 70.5  (66.7; 74.3) | 77.0  (73.8; 80.2) |
|  | Return to formal full-time work ability after one year (%)  (only in patients <66 years) | 87.3  (86.1; 88.4) | 88.2  (86.1; 90.2) | 86.8  (85.4; 88.3) | 72.1  (68.1; 76.0) | 74.1  (67.1; 81.0) | 71.1  (66.3; 76.0) |
| Resource use | Initial inpatient stay (days) | 13.2  (13; 13.4) | 13.4  (13.1; 13.7) | 13.1  (12.8; 13.4) | 24.1  (23.0; 25.2) | 22.1  (20.8; 23.4) | 25.8  (24.1; 27.6) |
|  | Inpatient stay first year (days) | 20.0  (19.6; 20.4) | 20.1  (19.6; 20.6) | 19.9  (19.4; 20.5) | 36.2  (34.5; 38) | 34.5  (32.1; 36.8) | 37.7  (35.2; 40.3) |
|  | Outpatient care first year (visits) | 20.3  (19.9; 20.6) | 19.1  (18.6; 19.5) | 21.4  (20.9; 21.8) | 21.4  (20.3; 22.6) | 19.4  (17.8; 21.1) | 23.2  (21.5; 24.8) |
|  | Net days of sick-leave/disability pension (patients <66 years) | 176.0  (171.3; 180.7) | 193.0  (185.3; 200.8) | 166.4  (160.5; 172.2) | 219.0  (207.1; 230.9) | 224.2  (204; 244.4) | 216.2  (201.4; 230.9) |
|  | Added home-help services (hours) | 106.0  (100.3; 111.8) | 128.2  (118.9; 137.5) | 87.2  (80.1; 94.3) | 180.4  (152.3; 208.4) | 209.9  (163.6; 256.2) | 157.9  (123.3; 192.6) |
|  | Transfer to special housing (%) | 11.2  (10.7; 11.6) | 14.4  (13.6; 15.1) | 8.3  (7.7; 8.8) | 15.6  (14; 17.2) | 20.8  (18.1; 23.5) | 11.2  (9.3; 13.1) |
| Process measures | Thrombolysis (%) | 7.1  (6.8; 7.4) | 6.2  (5.8; 6.7) | 8.0  (7.5; 8.5) |  |  |  |
|  |  |  |  |  |  |  |  |
|  | Thrombectomy (%) | 1.0  (0.9; 1.2) | 0.8  (0.6; 0.9) | 1.3  (1.1; 1.5) |  |  |  |
|  |  |  |  |  |  |  |  |
|  | High three-month patient satisfaction (%) | 51.7  (50.9; 52.5) | 50.6  (49.4; 51.7) | 52.7  (51.7; 53.8) | 47.6  (45.1; 50.1) | 45.9  (42.2; 49.6) | 48.9  (45.5; 52.3) |

**Table S2. Analysis population sizes and p-values from multivariate regression analyses**

|  | Sex as predictor for IS | | Sex as predictor for ICH | |
| --- | --- | --- | --- | --- |
|  | n | p-value | n | p-value |
| 1-year survival | 22,761 | <0.001*** | 2,975 | <0.001*** |
| 1-year recurrent stroke | 17,052 | 0.59 | 1,770 | 0.32 |
| Good 1-year functioning (approximated mRS 0-2) | 12,345 | <0.001*** | 1,148 | 0.82 |
| Good 1-year general health | 12,189 | 0.19 | 1,170 | 0.79 |
| Return to formal full-time work ability | 2,876 | 0.28 | 465 | 0.13 |
| Initial inpatient stay | 20,575 | <0.001*** | 2,124 | <0.005** |
| Inpatient days first year | 17,716 | <0.001*** | 1,841 | 0.46 |
| Outpatient visits first year | 17,690 | 0.92 | 1.839 | 0.89 |
| Net days of sick-leave/disability pension | 3,960 | <0.001*** | 581 | 0.10 |
| Added home-help services | 15,579 | 0.84 | 1,554 | 0.90 |
| Transfer to special housing | 21,756 | <0.05* | 2,816 | 0.26 |
| Thrombolysis | 22,564 | 0.12 |  |  |
| Thrombectomy | 22,714 | 0.45 |  |  |
| High three-month patient satisfaction | 15,592 | 0.15 | 1,473 | 0.63 |
